# Psychometric Properties and Measurement Invariance of the Childhood Trauma Questionnaire (Short Form) Across Genders, Time Points and Presence of Major Depressive Disorder Among Chinese Adolescents

**DOI:** 10.3389/fpsyg.2022.816051

**Published:** 2022-04-11

**Authors:** Xin Wang, Fengjiao Ding, Chang Cheng, Jiayue He, Xiang Wang, Shuqiao Yao

**Affiliations:** ^1^Medical Psychological Center, The Second Xiangya Hospital, Central South University, Changsha, China; ^2^Medical Psychological Institute, Central South University, Changsha, China; ^3^China National Clinical Research Center on Mental Disorders (Xiangya), Changsha, China

**Keywords:** CTQ, child maltreatment, measurement invariance, depression, Chinese adolescents

## Abstract

**Purpose:**

The Childhood Trauma Questionnaire-Short Form (CTQ-SF) is a widely used self-report tool designed to assess juveniles’ experiences of abuse and neglect. The current study examined the psychometric properties, particularly measurement invariance of the CTQ-SF in Chinese non-clinical adolescents and adolescents with major depressive disorder (MDD).

**Methods:**

Participants included 1,507 high school students (non-clinical sample) from Hunan Province and 281 adolescent patients with major depressive disorder (MDD sample) from The Second Xiangya Hospital. We examined the reliability and validity of CTQ-SF, confirm the five-factor model of the CTQ-SF. Multiple-group confirmatory factor analysis (CFA) was used to examine the measurement invariance across genders, presence of depression, and over time.

**Results:**

The CTQ-SF had good internal consistency in a non-clinical sample (Cronbach’s α = 0.85) and MDD sample (Cronbach’s α = 0.86). Good test–retest reliability (ICC = 0.72) and Adequate validity were also observed. Good fit of the five-factor CTQ-SF model was confirmed in both samples. Multiple-group CFA confirmed that the CTQ-SF had the scalar invariance across genders and the presence of MDD, as well as over time.

**Conclusion:**

The CTQ-SF is an effective and reliable tool for assessing child maltreatment in Chinese adolescents (non-clinical sample and MDD sample). The results suggest that the horizontal and longitudinal invariance of CTQ-SF are strongly established, which means CTQ-SF can be meaningfully used to compare outcomes among Chinese adolescents (non-clinical sample and MDD sample). The experience of child maltreatment, especially neglect (emotional and physical), was found to be common in Chinese adolescents.

## Introduction

Child maltreatment (CM) is defined as the abuse and neglect of people under the age of 18 years. CM includes physical, sexual, and psychological abuse or neglect and it may occur in a home, at school, or in the communities (Fu et al., 2018; He et al., 2019). A meta-analysis of data collected from Chinese undergraduates showed a pooled prevalence of CM of 64.7%, with the following pooled estimates by CM types: Physical Abuse, 17.4%; Emotional Abuse, 36.7%; Sexual Abuse, 15.7%; Physical Neglect, 54.9%; and Emotional Neglect, 60.0% (Fu et al., 2018). CM is a strong social and environmental risk factor for disease, disability, and death at all stages of life (Viola et al., 2016). It not only affects people’s social function but also causes a heavy economic burden (Fang et al., 2015; Carr et al., 2020). Many researchers have also reported that CM has a lasting impact on mental health, including increasing one’s risk of mental illness, worsening the severity and types of psychiatric symptoms one experiences, and increasing the frequency of comorbidity, especially depression (Pearlman and Courtois, 2005; Draper et al., 2008; Berthelot et al., 2019; Stanton et al., 2020). Indeed, CM has been established as a major risk factor for depression in adolescents and adults (De Bellis et al., 2019; Chen et al., 2021).

The Childhood Trauma Questionnaire (CTQ) is a 70-item self-administered inventory developed by Bernstein et al. (2003) with the aim of providing a reliable and effective retrospective assessment of child abuse and neglect. CTQ showed a stable five-factor structure in adult substance abusers and adolescent psychiatric inpatients (Bernstein et al., 2003) and good reliability and validity in a Canadian undergraduate sample and an American community sample (Scher et al., 2001; Paivio and Cramer, 2004). The CTQ has since been translated into many languages (Persian, German, Portuguese, etc.) and has been shown to have good measurement properties across national cultures (Grassi-Oliveira et al., 2006; Garrusi and Nakhaee, 2009; Wingenfeld et al., 2010). Because the original 70-item version of the CTQ covers a large number of topics and takes a long time to complete, it can be burdensome for respondents to complete. Bernstein and colleagues thus developed an abbreviated version, the CTQ-Short Form (CTQ-SF), to enable fast screening for abuse history (Bernstein et al., 2003). The CTQ-SF included 25 clinical items and 3 validity items, including five clinical subscales (Physical Abuse, Sexual Abuse, and Emotional Abuse, Physical Neglect, and Emotional Neglect) and a three-item minimization/denial validity scale for detecting the underreporting of abuse. A five-factor structure analogous to the structure of the original CTQ was found to provide a good fit for the CTQ-SF in their four test groups with satisfactory measurement invariance among the four samples (Bernstein et al., 2003). Versions of the CTQ-SF produced in several languages (Spanish, German, Chinese, and Korean, among others) have since been shown to have good reliability and validity. Notably, translated versions of the CTQ-SF have been shown to have a stable five-factor structure across clinical and non-clinical samples, including four clinical samples from Denmark (Kongerslev et al., 2019), an Italian student sample (Sacchi et al., 2017), and a Chinese undergraduate sample (He et al., 2019). In addition, the CTQ-SF has been shown to have scalar equivalence across gender groups in Nigerian teenaged and Chinese college student samples, as well as partial weak equivalence across gender and race groups of drug-abusing adults (Thombs et al., 2007; He et al., 2019; Aloba et al., 2020).

Measurement invariance refers to the notion that scores obtained on an instrument for individuals belonging to different groups are comparable because the scores have a consistent meaning across the groups (Meredith, 1993). Measurement invariance of the CTQ-SF has not yet been studied in Chinese adolescents. Adolescence is a critical period of development characterized by vulnerability to influences from and changes in one’s environment, including trauma-associated influences and changes (Crews et al., 2007). A large number of studies have shown that the effect of CM on depression is mainly in adolescence. It elevates the risks of individual functional structural abnormalities and depression in adolescence by affecting the development process of the nervous system (Moretti and Craig, 2013; Teicher and Samson, 2016; Dow-Edwards et al., 2019). Besides, traumatic experiences in childhood also affect the impacts of psychological treatment and rehabilitation in adolescents with depression. Compared with adolescents without CM, adolescents with CM show slower improvement in the severity of depressive symptoms in response to receiving psychotherapy (Waldron et al., 2019). Therefore, the research on the experience of CM in adolescence is quite necessary and deserves more attention (De Bellis et al., 2019; Gruhn and Compas, 2020; LeMoult et al., 2020). A standard and effective measurement instrument for screening and early intervention of CM experience at this age is essential, which is why we would like to validity CTQ-SF, one of the most widely used measurement tool, to assess the experiences of abuse and neglect in healthy and depressive adolescents. In addition, CM is greatly influenced by cultural differences. There are significant differences in the prevalence of different forms of CM between Eastern and Western cultures (Stoltenborgh et al., 2015), As far as we know, up to now, little research reporting the measurement invariance of CTQ-SF among adolescents, with only one study demonstrating the gender equivalence in Nigerian adolescents (Aloba et al., 2020).

Therefore, the main objective of the present study is to examine the psychometric properties, particularly measurement invariance of CTQ-SF among Chinese non-clinical and MDD adolescents. First, we investigate the prevalence of CM in non-clinical samples. Then examine the reliability, validity and factor structure of the scale among non-clinical and MDD samples. Lastly, we explored the measurement invariance cross-gender, time, and psychiatric diagnosis of MDD, investigating the applicability of the childhood trauma questionnaire in adolescents comprehensively. These findings are especially valuable given that adolescents often show greater gender and time variability than adults, which provides a measurement basis for the usage of CTQ-SF in the study of childhood abuse in adolescents.

## Materials and Methods

### Participants

The study was approved by the Ethics committee of Second Xiangya Hospital, Central South University. Clinical (patients with MDD) and Non-clinical (senior high school students) samples were recruited from 2015 through 2019. The age range of all participants was 15–17 years old, and all participants and their parents provided written informed consent before completing the study questionnaires.

#### Non-clinical Sample

Adolescents were recruited through posters and advertisements from two senior high schools in Hunan Province. Data collection was carried out by two trained researchers and participants with a history of mental disorders were excluded. A total of 1,600 high school students took questionnaire surveys, of which 93 were omitted for being incomplete. The remaining 1,507 valid questionnaires were obtained from 681 male (45.2%) and 826 (54.8%) female students. The mean (standard deviation, SD) age of the non-clinical sample was 16.10 (0.84) years. To examine test-retest reliability and measurement invariance of CTQ-SF, 4 weeks following the initial assessment, a CTQ-SF retest was conducted to a subset of 1,000 high school participants [426 males (45.2%) and 574 females (54.8%)].

#### Clinical Major Depressive Disorder Sample

Outpatients diagnosed with MDD were recruited by two psychiatrists from Second Xiangya Hospital of Central South University and were assessed with Kiddie Schedule for Affective Disorders and Schizophrenia (K-SADS). The exclusion criteria for the clinical sample were: inability to understand the questions well; currently or previously meeting the DSM-IV-TR criteria for any disorder except MDD (i.e., mental disorder comorbidity); a history of alcohol/substance abuse. The final MDD sample consisted of 281 participants. The mean (SD) age of the MDD sample participants was 16.12 (0.82) years.

### Instruments

#### Childhood Trauma Questionnaire-Short Form

Child maltreatment exposure was assessed with the 28-item Chinese version of the Childhood Trauma Questionnaire-Short Form (CTQ-SF), which was introduced and translated from Bernstein’s original English version (Bernstein et al., 2003) by Zhao (2004). The questionnaire consists of 28 items (25 clinical items and 3 validity items), including five dimensions: Physical Abuse, Physical Neglect, Emotional Abuse, Emotional Neglect, and Sexual Abuse. Each item on the questionnaire was rated on a 5-point Likert scale (score range, 1–5). The scores of each of the five subscales of the CTQ-SF ranged from 5 to 25, and total scores for the CTQ-SF ranged from 25 to 125. Participants with scores higher than any of the following subscale thresholds were considered to have experienced CM: Emotional Neglect ≥ 10; Physical Neglect ≥ 8; Emotional Abuse ≥ 9; Physical Abuse ≥ 8; and Sexual Abuse ≥ 6(Pham et al., 2021).

#### Beck Depression Inventory

Beck Depression Inventory (BDI) was developed by Beck et al. (1961) in the United States, is a well-established self-rating depression scale with good psychometric properties (Hovens et al., 2009). It consists of 21 items, each of which includes four statements describing varying levels of intensity of depressive symptoms. For each item, respondents choose the statement that best approximates their feelings in the past week, and the scoring value of the statements within each item are 0, 1, 2, and 3, respectively. BDI total scores range from 0 to 63, with higher scores indicating more severe depressive symptoms.

#### State-Trait Anxiety Inventory

The Spielberger State-Trait Anxiety Inventory (STAI) (Spielberger et al., 1983) is a 40-item instrument used to measure transient (state) and enduring (trait) levels of anxiety, each of which is determined on its 20-item subscale. Specifically, the State Anxiety Inventory (SAI) subscale is used to evaluate transitory emotional responses to stress-inducing situations that involve unpleasant feelings of tension and apprehensive thoughts. Meanwhile, the Trait Anxiety Inventory (TAI) subscale is used to assess the respondent’s relatively stable emotional disposition (Kennedy et al., 2001). Each item on the questionnaire was rated on a 4-point Likert scale (score range, 1–4). with a higher total score indicating greater anxiety. The STAI has been shown to have good psychometric properties (Lee et al., 2017).

#### Schedule for Affective Disorder and Schizophrenia for School-Age Children

The Schedule for Affective Disorder and Schizophrenia for School-Age Children (K-SADS) is a diagnostic semi-structured interview questionnaire designed to assess current and past psychiatric episodes in school-aged children and adolescents based on DSM-VI diagnostic criteria. The questionnaire consists of two parts. The first part is a non-structured guided interview used to collect basic demographic data, family history, past medical history, and growth history. The second part constitutes a psychometric evaluation during which data describing the frequency, severity, and features of various psychiatric symptoms are collected. The K-SADS has been demonstrated previously to be suitable for use with Chinese adolescents (Yang et al., 2010), and it has been applied extensively in clinical child psychology research. In this study, the scale was used mainly to screen for and diagnose depression. At the end of the psychometric evaluation interview, participants with MDD were asked questions intended to detect signs of mania and psychosis to detect or exclude bipolar and psychotic disorders.

### Data Analysis

All primary data analyses were done in SPSS, version 22.0. Firstly, descriptive analyses were conducted to depict the participants’ performance on demographic characteristics and other study measures. And then investigated the prevalence of CM in the tow samples. Afterward, the reliability of the data is analyzed, Cronbach’s α values were calculated to determine the internal reliability of the CTQ-SF. Cronbach’s α value > 0.60 was considered acceptable (Cronbach, 1951; Devellis, 1991).

Intraclass correlation coefficient (ICC) was used to evaluate the test-retest reliability. In general, ICC values of 0.00 to 0.20 indicate slight reliability, 0.21 to 0.40 fair, 0.41 to 0.60 moderate,0.61 to 0.80 substantial, and ≥0.81 almost perfect reliability (Shrout and Fleiss, 1979). The Measurement Error is usually expressed as the mean difference between measured and true values. The Measurement Error of CTQ-SF is measured by Standard Error of Measurement (SEM), Minimal Detectable Change (MDC) and Limits of Agreement (LoA). SEM is estimated from the standard deviation (SD) of a sample of scores at baseline and a test–retest reliability index of the measurement instrument used [e.g., SEM = SD × √ (1–ICC)] (Furlan and Sterr, 2018). The MDC value might be considered as the minimum amount of change that needs to be observed at either group or individual level, to be considered a real change. MDC is estimated from SEM and 95% degree of confidence (e.g., MDC95 = 1.96 × √2 × SEM) (Furlan and Sterr, 2018). Scatter plot and 95% LoA of the difference between the CTQ-SF scores of the two measurements were drawn to reflect the distribution of the difference between the two measurements. Bland–Altman method was used to draw in MedCalc software (Bland and Altman, 1999). After that, the data were evaluated for validity. Since the strong connection between CM with depression and anxiety, the criterion-related validity was evaluated by analyzing the correlation between CTQ-SF with BDI and STAI. In order to further determine the influence of CM on depression and anxiety, stepwise multiple regression analysis (MRA) was conducted with depression and anxiety as dependent variables respectively and five dimensions of CM as independent variables. The effect size is based on Cohen’s *f*^2^ (Cohen, 1988; Selya et al., 2012).

In our study, we treated the CTQ-SF score as interval level data (a scale which Likert-type items possess 5–15 levels could be treated as interval), the Kolmogorov–Smirnov test shows that each item is significantly different from the normal distribution (*P* < 0.001), so the confirmatory factor analysis (CFA) was performed with maximum likelihood estimation (MLM) in M-plus 7 to assess how well our dataset adhered to the five-factor model of the CTQ-SF (Satorra and Bentler, 1994; Lubke and Muthn, 2004; Finney and Distefano, 2013). Since the chi-square test is easily affected by sample size, even if the difference is small, as long as the sample size is large, it will show a significant difference (Cheung and Rensvold, 1999). Considering our relatively large sample size, to evaluate the model of the CTQ-SF, the Tucker–Lewis index (TLI), comparative fit index (CFI), and root mean square error of approximation (RMSEA) were used with the following common criteria for model acceptability: RMSEA ≤ 0.08, SRMR ≤ 0.08, TLI ≥ 0.90, and CFI ≥ 0.90 (Hu and Be Ntler, 1998).

To ensure that the group differences in CTQ-SF scores were not due to measurement artifacts, multi-group CFAs were used to examine measurement invariance(MI) across samples (Wang et al., 2013). In our study, four procedures were conducted to assess CTQ-SF measurement invariance across gender among non-clinical sample. Firstly, configural invariance concerns whether two groups have the same factor structure (Horn and McArdle, 1992). It was tested by restricting the measurement model to be equal across groups and allowing all parameters (factor loadings, intercepts) to be freely estimated within groups (Kline et al., 2011). Secondly, metric invariance testing whether the strengths of the relationships are the same across two groups to examines differential responding to items, which is done by constraining the unstandardized factor loadings of each indicator to be equal across two groups (Horn and McArdle, 1992; Avila et al., 2020). Thirdly, scalar invariance examines whether the group differences in CTQ-SF scores are due to group differences at the latent level or factor mean (Meitinger, 2017). Based on the metric invariance model, another restriction is added that constrains the intercepts of the observed variables to be the same across groups. Lastly, strict invariance is the most restrictive level of invariance which requires equal factor loadings, intercepts, and residual variances. Each process is executed based on satisfying the previous condition. Then we examine the measurement invariance across the presence of depression and time using the same processes. Measurement invariance was considered to be established when the following criteria were satisfied: ΔCFI ≤ 0.01, ΔTLI ≤ 0.01, and ΔRMSEA ≤ 0.015 (Cheung and Rensvold, 2002; Chen, 2007).

After that, we compared the differences in CTQ-SF mean scores between non-clinical and MDD groups and between men and women groups among the two samples. The effect size was based on the Cohen’s *d*, in which effect size was either small (0.2 < Cohen’s *d* < 0.5), medium (0.5 < Cohen’s *d* < 0.8), or large (Cohen’s *d* > 0.8) (Cohen, 1992).

## Results

### Descriptive Statistics and Prevalence

The demographic characteristics of non-clinical and MDD samples were summarized in Table 1. Notably, age and education did not differ significantly between the two samples and. However, representation of the sexes did differ between two samples, with girls representing a much larger proportion of the MDD sample than of the non-clinical sample, consistent with the previously established trend of there being a higher incidence of depression of females than males in China (Gu et al., 2013; Kuehner, 2017). Obviously, these two groups differed in measures of depression and anxiety.

**TABLE 1:** Demographic information of the Non-clinical and MDD samples.

Variable	Non-clinical sample (*N* = 1,507)	MDD sample (*N* = 281)	Statistic	*P* (two-tailed)
**Demographics, mean score (SD)**				
Age, years	16.10 (0.84)	16.12 (0.82)	*t* = –0.41	0.684
Gender (M/F)	826/681	218/63	χ^2^ = 50.54	<0.001
Years of education	10.23 (0.68)	10.18 (0.65)	*t* = 1.12	0.264
**Psychometrics, mean score (*SD*)**				
BDI score	7.76 (5.46)	27.47 (7.30)	*t* = –43.04	<0.001
SAI score	42.53 (9.44)	58.19 (8.77)	*t* = –25.79	<0.001
TAI score	44.21 (7.89)	57.87 (7.34)	*t* = –26.93	<0.001

*Notes. All data, except gender ratios, are presented in the form of mean (standard deviation). BDI, Beck Depression Inventory; CTQ-SF, Childhood Trauma Questionnaire-Short Form; MDD, major depressive disorder; M/F, ratio of number of males to females; S/TAI, State/Trait Anxiety Inventory.*

Tables 2–4 shows the prevalence of CTQ-SF among different groups. Of the 1,507 non-clinical adolescents surveyed, 78.10% had experienced at least one type of CM before the age of 18 years. The prevalence of MDD sample is significantly higher than that of non-clinical samples in all dimensions except Sexual Abuse, and the cumulative prevalence of CM was also significantly higher than that of non-clinical samples except five types. In terms of gender, in non-clinical samples, the prevalence of Physical Neglect, Physical Abuse and Sexual Abuse in boys is significantly higher than that in girls, and the cumulative prevalence of CM was also significantly higher than that of girls. In MDD samples, the prevalence of Emotional Neglect in boys is significantly higher than that in girls, with no significant differences in other dimensions.

**TABLE 2:** Prevalence of CTQ-SF between different samples.

Type of maltreatment	Non-clinical sample (*n* = 1057) % (95% CI)	MDD sample (*n* = 281) % (95% CI)	χ 2
Emotional abuse	17.25 (15.34–19.16)	40.93 (35.14–46.71)	80.076^***^
Physical neglect	60.05 (57.58–62.53)	75.09 (70.00–80.18)	22.825^***^
Emotional neglect	58.26 (55.78–60.75)	71.89 (66.60–77.17)	18.382^***^
Physical abuse	6.44 (5.20–7.78)	13.88 (9.81–17.95)	18.666^***^
Sexual abuse	13.87 (12.12–15.62)	13.88 (9.81–17.95)	<0.001
Any type of maltreatment (≥ 1)	78.10 (76.01–80.19)	91.81 (88.06–95.43)	28.107^***^
Any type of maltreatment (≥ 2)	51.76 (49.23–54.28)	69.04 (59.72–72.39)	28.518^***^
Any type of maltreatment (≥ 3)	17.92 (17.79–23.91)	37.01 (31.33–42.69)	52.200^***^
Any type of maltreatment (≥ 4)	6.24 (5.02–7.46)	15.44 (11.38–19.93)	29.512^***^
Any type of maltreatment (≥5)	1.86 (1.18–2.54)	2.14(0.43–3.84)	0.098

CTQ-SF, Childhood Trauma Questionnaire-Short Form; MDD, major depressive disorder; CI, confidence interval. *p < 0.05, **p < 0.01, ***p < 0.001.

**TABLE 3:** Prevalence of CTQ-SF in non-clinical sample.

Type of maltreatment	Non-clinical Sample			
	Male (*n* = 681) % (95% CI)	Female (*n* = 826) % (95% CI)	Total (*n* = 1057) % (95% CI)	χ ^2^
Emotional abuse	17.47 (14.62–20.33)	17.07 (14.50–19.64)	17.25 (15.34–19.16)	0.043
Physical neglect	65.49 (61.91–69.07)	55.57 (52.17–58.96)	60.05 (57.58–62.53)	15.321^***^
Emotional neglect	59.47 (55.77–63.17)	57.26 (53.88–60.64)	58.26 (55.78–60.75)	0.748
Physical abuse	10.43 (8.12–12.73)	3.15 (1.95–4.34)	6.44 (5.20–7.78)	27.544^***^
Sexual abuse	16.15 (13.38–18.92)	11.99 (9.77–14.21)	13.87 (12.12–15.62)	5.426*
Any type of maltreatment (≥ 1)	80.76 (77.80–83.73)	75.91 (72.99–78.83)	78.10 (76.01–80.19)	5.146*
Any type of maltreatment (≥ 2)	55.95 (52.21–59.69)	48.31 (52.21–59.69)	51.76 (49.23–54.28)	8.730^**^
Any type of maltreatment (≥ 3)	20.85 (17.79–23.91)	15.50 (17.79–23.91)	17.92 (17.79–23.91)	7.279^**^
Any type of maltreatment (≥ 4)	8.52 (17.79–23.91)	4.36 (17.79–23.91)	6.24 (5.02–7.46)	11.037^**^
Any type of maltreatment (≥ 5)	2.94 (1.67–4.21)	0.97 (1.67–4.21)	1.86 (1.18–2.54)	7.931**

CTQ-SF, Childhood Trauma Questionnaire-Short Form; CI, confidence interval. *p < 0.05, **p < 0.01, ***p < 0.001.

**TABLE 4:** Prevalence of CTQ-SF in MDD sample.

Type of maltreatment	MDD sample			
	Male (*n* = 63) % (95% CI)	Female (*n* = 218) % (95% CI)	Total (*n* = 281) % (95% CI)	χ 2
Emotional abuse	36.51 (24.29–48.73)	42.20 (35.59–48.81)	40.93 (35.14–46.71)	0.655
Physical neglect	79.37 (69.09–89.64)	73.85 (69.09–89.64)	75.09 (70.00–80.18)	0.794
Emotional neglect	84.13 (74.85–93.40)	68.35 (74.85–93.40)	71.89 (66.60–77.17)	6.021*
Physical abuse	11.11 (3.13–19.09)	14.68 (9.94–19.41)	13.88 (9.81–17.95)	0.521
Sexual abuse	20.63 (70.00–80.18)	11.93 (70.00–80.18)	13.88 (9.81–17.95)	3.101
Any type of maltreatment (≥1)	92.06 (85.20–98.93)	91.74 (88.06–95.43)	91.81 (88.06–95.43)	0.007
Any type of maltreatment (≥2)	79.37 (69.09–89.64)	66.06 (59.72–72.39)	69.04 (59.72–72.39)	4.051*
Any type of maltreatment (≥3)	42.86 (30.29–55.42)	35.32 (28.93–41.72)	37.01 (31.33–42.69)	1.191
Any type of maltreatment (≥4)	17.46 (7.82–27.10)	15.14 (10.34–19.93)	15.44 (11.38–19.93)	0.200
Any type of maltreatment (≥5)	0.00	2.75 (0.56–4.94)	2.14 (0.43–3.84)	1.772

CTQ-SF, Childhood Trauma Questionnaire-Short Form; MDD, major depressive disorder; CI, confidence interval. *p < 0.05, **p < 0.01, ***p < 0.001.

### Reliability

The Cronbach’s α of non-clinical sample (range, 0.48–0.85) and MDD sample (range, 0.54–0.89), 4-week test–retest reliability (ICC) (range, 0.44–0.72) values of non-clinical sample are reported in Table 5. Briefly, adequate reliability was observed for the total scale and all subscales, except the Physical Neglect subscale among two samples. The subscales with the highest Cronbach’s α were Emotional Neglect for the non-clinical sample and Sexual Abuse for the MDD sample. The Physical Neglect subscale had the lowest Cronbach’s α among the two groups. The ICC for the CTQ-SF total scale was 0.72, which showed an acceptable test–retest reliability.

**TABLE 5:** Cronbach’s α values and ICC for CTQ-SF and subscales.

Scale	Non-clinical sample	MDD sample	ICC
CTQ-SF	0.85	0.86	0.72
Emotional neglect	0.82	0.82	0.70
Physical neglect	0.48	0.54	0.59
Sexual abuse	0.76	0.89	0.44
Emotional abuse	0.68	0.77	0.63
Physical abuse	0.77	0.84	0.54

*CTQ, Childhood Trauma Questionnaire-Short Form; MDD, major depressive disorder; ICC, intraclass correlation coefficient.*

### Measurement Error of Childhood Trauma Questionnaire-Short Form

**FIGURE 1:**
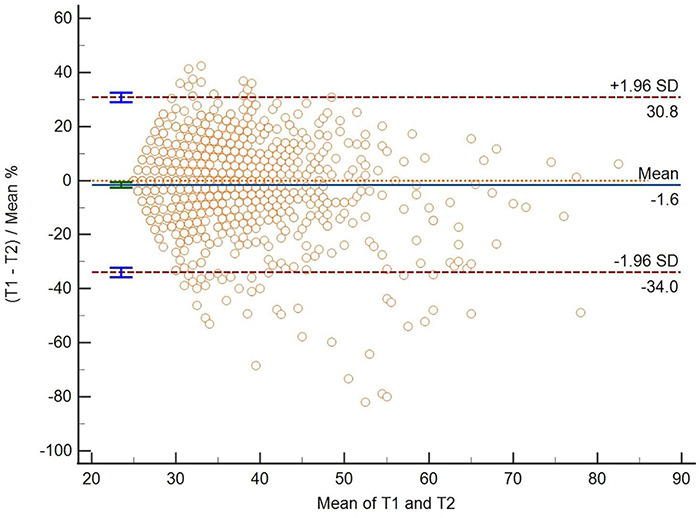
The bias line and random error lines forming the 95% limits of agreement are presented on the plot. T1, the CTQ-SF score of the first measurement; T2, the CTQ-SF score of the second measurement; SD, standard deviation.

The estimates of SEM were 4.48, and MDC_95_ were 12.41. The mean deviation of CTQ-SF scores before and after the two measurements was –1.6, and the 95% Limits of Agreement (mean ± 1.96 standard deviation) ranged from -34.0 to 30.8. The results of the two measurements of CTQ-SF scale were mostly distributed on both sides of the mean, and a small part exceeded the range, as shown in Figure 1.

### Validity

Intercorrelation among the CTQ-SF and its subscale were shown in Table 6. In the non-clinical sample, all correlations were positive and significant (range, 0.156–0.852; all *p* < 0.05). In the MDD sample, the correlation coefficients ranged from 0.083 to 0.810 and all correlations were positive and significant (*p* < 0.05) except the correlation between Sexual Abuse and Emotional Neglect.

**TABLE 6:** Correlations among total and subscale scores of CTQ-SF.

	CTQ-SF	Emotional neglect	Physical neglect	Sexual abuse	Emotional abuse

**Non-clinical sample**					
EN	0.852**				
PN	0.735**	0.523**			
SA	0.387**	0.156**	0.166**		
EA	0.709**	0.414**	0.343**	0.272**	
PA	0.607**	0.342**	0.249**	0.347**	0.538**

**MDD sample**					

EN	0.810**				
PN	0.615**	0.396**			
SA	0.400**	0.083	0.197**		
EA	0.785**	0.487**	0.247**	0.282**	
PA	0.673**	0.375**	0.246**	0.315**	0.528**

*p < 0.05; **p < 0.01. CTQ-SF, Childhood Trauma Questionnaire-Short Form; MDD, major depressive disorder.

The Pearson correlations among the CTQ-SF total scale and its subscales with BDI, SAI, and TAI scores were reported in Table 7. In the non-clinical sample, all correlations were positive and significant (range, 0.105–0.412; all *p* < 0.05), with the highest bivariate correlation between total CTQ score with anxiety and depression. In the MDD sample, the highest bivariate correlations between CTQ-SF with anxiety and depression were Emotional Abuse. In the subscale of two samples, the correlations between Emotional Neglect with anxiety and depression were also meaningful. Table 8 presents the results of two MRA with regard to depression and anxiety. The results showed that in the non-clinical samples, Emotional Abuse, Emotional Neglect, Physical Neglect, Sexual Abuse entered the regression model of depression and anxiety, which had significant direct positive effects on depression and anxiety; in the clinical samples, Emotional Abuse, Emotional Neglect entered the regression model of depression, which had significant direct positive effects on depression; Emotional Abuse entered the regression model of anxiety, which had a significant direct positive effect on anxiety.

**TABLE 7:** Correlations among total and subscale scores of CTQ-SF with other scales.

	CTQ-SF	Emotional neglect	Physical neglect	Sexual abuse	Emotional abuse	Physical abuse

**Non-clinical sample**						
SAI	0.343**	0.308**	0.231**	0.133**	0.260**	0.181**
TAI	0.392**	0.360**	0.282**	0.105**	0.298**	0.177**
BDI	0.412**	0.328**	0.261**	0.172**	0.391**	0.234**

**MDD sample**						

SAI	0.265**	0.221**	0.061	0.081	0.270**	0.209**
TAI	0.313**	0.292**	0.041	0.098	0.337**	0.203**
BDI	0.410**	0.369**	0.094	0.127*	0.430**	0.248**

*p < 0.05; **p < 0.01. CTQ-SF, Childhood Trauma Questionnaire-Short Form; MDD, major depressive disorder; BDI, Beck Depression Inventory; S/TAI, State/Trait Anxiety Inventory.

**TABLE 8:** Multiple regression analysis on symptoms of depression and anxiety for non-clinical sample and MDD sample.

	Depression				Anxiety			
	Factor	β (standardized)	*t*	Cohen’s *f*^2^	Factor	β (standardized)	t	Cohen’s *f*^2^
Non-clinical sample	EA	0.282	10.656***	0.181	EA	0.133	4.829***	0.023
	EN	0.167	5.877***	0.042	EN	0.211	7.094***	0.105
	PN	0.069	2.504*	0.005	PN	0.066	2.284*	0.005
	SA	0.060	2.478*	0.004	SA	0.052	2.069*	0.002
model		*F*(4,1502) = 90.368; *P* < 0.001				*F*(4,1502) = 51.896; *P* < 0.001		
Explained variance(*R*^2^)		*R*^2^ = 0.20				*R*^2^ = 0.12		
MDD sample	EA	0.328	5.414***	0.224	EA	0.270	4.690***	0.079
	EN	0.210	3.455**	0.040				
model		*F*(2,278) = 38.938; *P* < 0.001				*F*(1,279) = 21.996; *P* < 0.001		
Explained variance(*R*^2^)		*R*^2^ = 0.21				*R*^2^ = 0.07		

MDD, major depressive disorder; EA, Emotional Abuse; EN, Emotional Neglect; PN, Physical Neglect; SA, Sexual Abuse. *p < 0.05, **p < 0.01, ***p < 0.001.

### Goodness of Fit of the Five-Factor Model of the Childhood Trauma Questionnaire-Short Form

The goodness of fit indices obtained from single-group CFA of the CTQ-SF is reported in Table 9. All fit indices met the criteria for an acceptable model fit in the non-clinical sample and female subgroup of the non-clinical sample. However, the model had poor fit indices in the MDD sample, the male subgroup of the non-clinical sample, the initial test, and the retest sample. The subsequent modification of the model according to the modification indices, and the acceptable fitting index were obtained for each sample. These results showed that the modified five-factor model could be used as an initial model for the following measurement invariance tests.

**TABLE 9:** Fit indices summary for the five-factor CTQ-SF model.

Sample	S-Bχ 2	CSF	df	CFI	TLI	RMSEA (90%CI)	SRMR
Non-clinical (*N* = 1507)	676.333*	2.199	265	0.914	0.903	0.032(0.029–0.035)	0.049
Males (*N* = 681)	477.553*	1.859	264	0.909	0.920	0.034(0.029–0.039)	0.053
Females (*n* = 826)	479.799*	2.103	265	0.906	0.917	0.031(0.027–0.036)	0.055
MDD(N = 281)	418.796*	1.443	264	0.914	0.903	0.046(0.037–0.054)	0.066
Test, Time1 (*N* = 1000)	545.403*	2.054	264	0.921	0.910	0.033(0.029–0.037)	0.055
Retest, Time2 (*N* = 1000)	691.306*	2.298	263	0.903	0.915	0.040(0.037–0.044)	0.062

*p < 0.05. MDD, major depressive disorder; Time 1: the first test; Time 2: retest four weeks later; Bχ2, Chi-square test of model fit; CSF: Scaling Correction Factor for MLM; df, degrees of freedom; CFI, comparative fit index; TLI, Tucker–Lewis index; RMSEA, root mean square error of approximation; SRMR, standardized root mean square residual; CI, confidence interval.

### Measurement Invariance of the Childhood Trauma Questionnaire-Short Form

Multiple-group CFA was performed to examine the measurement invariance of the CTQ-SF across genders among the non-clinical sample. Configural invariance examined whether the pattern of latent variables was the same in males and females. All fit indices (CFI = 0.926, TLI = 0.915, RMSEA = 0.031, SRMR = 0.055) were acceptable after modified and the configural invariance was established. Then the configural model was used as a baseline model for the subsequent analysis. Metric invariance examining whether factors are loaded similarly across males and females with other variables estimated freely, yielded acceptable fit index values (CFI = 0.918, TLI = 0.910, RMSEA = 0.032, SRMR = 0.066) as well as acceptable change in index values (ΔCFI = 0.008, ΔTLI = 0.005, and ΔRMSEA = 0.001). Thus, metric invariance was established. Subsequently, in scalar invariance testing with the intercept of the two groups is set to be equal based on the previous model, acceptable fit index values (CFI = 0.910, TLI = 0.905, RMSEA = 0.033, SRMR = 0.067) and acceptable change in index values(ΔCFI = 0.008, ΔTLI = 0.005, and ΔRMSEA = 0.001) were again obtained. Thus, scalar invariance across genders was established. In strict invariance testing with the residual variances of the two groups are set to be equal on the basis of the previous model. The model did not meet the requirements and strict invariance was not established.

Then we examine measurement invariance of CTQ-SF across non-clinical sample and MDD sample following the same steps delineated above for gender. The indices for configural, metric, scalar invariance models and the change in fit index values were acceptable. The indices for strict invariance were not acceptable thus the strict invariance was not established. At last, measurement invariance of CTQ-SF across the test and 4-week retest time points were examined and the configural, metric, scalar invariances were established. The indices of the strict invariance model did not meet the requirements and strict invariance was not established. The CFI, TLI, RMSEA, and SRMR fit index values obtained for all successive models developed during multiple-group CFA, and associated changes in index values obtained between successive models, are reported in Table 10.

**TABLE 10:** Measurement invariance of the CTQ-SF.

Model	S-Bχ 2	CSF	df	CFI	TLI	RMSEA(90%CI)	SRMR	Comparison	Δ CFI	Δ TLI

**Measurement invariance across genders**										
(A) Model 1	995.378*	1.989	530	0.911	0.900	0.034(0.031–0.037)	0.055		-	-
(B) Model 1_*modified*_	915.945*	1.996	526	0.926	0.915	0.031(0.028–0.035)	0.055		-	-
(C) Model 2	973.708*	2.081	546	0.918	0.910	0.032(0.029–0.036)	0.066	C vs. B	–0.008	–0.005
(D) Model 3	1038.149*	2.044	566	0.910	0.905	0.033(0.030–0.036)	0.067	D vs. C	–0.008	–0.005
(E) Model 4	1795.522*	2.294	591	0.770	0.767	0.052(0.049–0.055)	0.107	E vs. D	–0.140	–0.138

**Measurement invariance across presence of depression (Non-clinical vs. MDD)**										

(A) Model 1	1185.183*	1.833	530	0.909	0.897	0.037(0.034–0.040)	0.052		-	-
(B) Model 1_*modified*_	1119.572*	1.809	528	0.918	0.906	0.035(0.033–0.038)	0.052		-	-
(C) Model 2	1119.612*	1.873	548	0.920	0.913	0.034(0.031–0.037)	0.055	C vs. B	0.002	0.007
(D) Model 3	1151.214*	1.845	568	0.919	0.914	0.034(0.031–0.037)	0.055	D vs. C	–0.001	0.001
(E) Model 4	1531.782*	2.007	593	0.869	0.868	0.042(0.039–0.045)	0.061	E vs. D	–0.050	–0.046

**Measurement invariance across time (test vs. re-test)**										

(A) Model 1	2107.557*	1.942	1105	0.915	0.906	0.030(0.028–0.032)	0.052		-	-
(B) Model 2	2176.758 *	1.970	1125	0.911	0.903	0.031(0.029–0.032)	0.059	B vs. A	–0.004	–0.003
(C) Model 3	2230.155 *	1.952	1145	0.908	0.901	0.031(0.029–0.033)	0.059	C vs. B	–0.003	–0.002
(D) Model 4	2519.214*	2.024	1170	0.886	0.880	0.034(0.032–0.036)	0.069	D vs. C	–0.022	–0.021

*p < 0.05. The models were defined as follows: Model 1, configural invariance; Model 2, metric invariance; Model 3, strong invariance; and Model 4, strict invariance. Model 1_modified_ means the model after error covariances. Bχ2, Chi-square test of model fit; CSF: Scaling Correction Factor for MLM; df, degrees of freedom; CFI, comparative fit index; TLI, Tucker–Lewis index; RMSEA, root mean square error of approximation; SRMR, standardized root mean square residual. CI, confidence interval; Δ, difference.

### Comparisons of Childhood Trauma Questionnaire-Short Form Scores Across Groups

The mean CTQ-SF scale and subscale scores for groups with the associated statistical values are shown in Table 11. Compared to the non-clinical sample, the MDD sample had significantly higher CTQ-SF total scale scores and subscale scores except Sexual Abuse. There was no significant difference in Sexual Abuse subscale scores between the two samples. In terms of gender, except for Emotional Abuse, boys had significantly higher total scale scores and other subscale scores than girls in non-clinical sample. In MDD sample, boys scored significantly higher than girls in Emotional Neglect, and there was no significant difference in others.

**TABLE 11:** Childhood Trauma Questionnaire-Short Form mean score differences between groups.

	Sample		*t*	Sig.	Cohen’s *d*
	Non-clinical sample	MDD sample			
CTQ-SF	37.15 (8.51)	43.20 (10.88)	–0.8.84	<0.001	0.62
Emotional neglect	10.90 (4.22)	13.16 (4.78)	–7.41	<0.001	0.50
Physical neglect	8.64 (2.72)	9.73 (2.98)	–6.09	<0.001	0.38
Sexual abuse	5.33 (1.06)	5.42 (1.56)	–0.90	0.370	–
Emotional abuse	6.76 (2.28)	8.87 (3.84)	–8.91	<0.001	0.67
Physical abuse	5.51 (1.45)	6.02 (2.29)	–3.60	<0.001	0.27

	**Non-clinical sample**		***t***	**Sig.**	**Cohen’s *d***
	**Boys**	**Girls**			

CTQ-SF	38.23(9.30)	36.25(7.70)	4.43	<0.001	0.23
Emotional neglect	11.26(4.45)	10.61(4.01)	2.94	0.003	0.15
Physical neglect	9.04(2.86)	8.31(2.56)	5.21	<0.001	0.27
Sexual abuse	5.39(1.16)	5.28(0.97)	2.00	0.045	0.10
Emotional abuse	6.78(2.46)	6.75(2.12)	0.30	0.762	–
Physical abuse	5.75(1.75)	5.31(1.11)	5.74	<0.001	0.30

	**MDD sample**				
	**Boys**	**Girls**	***t***	**Sig.**	**Cohen’s *d***

CTQ-SF	45.24(11.18)	42.61(10.75)	1.69	0.092	–
Emotional neglect	14.46(4.84)	12.79(4.71)	2.47	0.014	0.35
Physical neglect	10.33(3.35)	9.56(2.85)	1.82	0.070	–
Sexual abuse	5.38(0.92)	5.43(1.71)	–0.20	0.839	–
Emotional abuse	8.89(4.15)	8.86(3.75)	0.048	0.962	–
Physical abuse	6.17(2.56)	5.98(2.21)	0.603	0.547	–

*CTQ-SF, Childhood Trauma Questionnaire-Short Form; MDD, major depressive disorder.*

## Discussion

This study examined the psychometric properties including measurement invariance of a Chinese version of the CTQ-SF in Chinese adolescents. Good internal consistency was confirmed for the CTQ-SF and all of its component subscales, except for Physical Neglect. Good test–retest reliability and Adequate validity were also observed. The previously established five-factor structure fits well with the data obtained with the present non-clinical sample as well as the subgroup of females and came to fit our other dataset satisfactorily following modifications. Multiple-group CFA confirmed that the CTQ-SF is strongly invariant across gender, time, and the presence of depression.

Our finding shows that in non-clinical sample, the prevalence of physical neglect, physical abuse, and sexual abuse in boys was significantly higher than that in girls, indicating that physical neglect, physical abuse, and sexual abuse are more common in boys. More than three-quarters of the adolescents had experienced some form of CM, more than 50 percent of adolescents have experienced at least two forms of CM, indicated that abuse and neglect are disturbingly common among Chinese adolescents, consistent with the findings of a meta-analysis published in Fu et al. (2018), which suggested that CM should be concerned by families, schools and the whole society. In the MDD sample, the prevalence of emotional abuse in boys was significantly higher than in girls, indicating that emotional abuse was more common in boys with adolescent MDD, with more than 90 percent of MDD adolescents having experienced at least some form of CM and about 70 percent of MDD adolescents having experienced at least two forms of CM. Suggesting that experiences of CM are more prevalent among MDD patients than healthy adolescents. Xie’s research also found that childhood maltreatment is more prevalent and more severe in patients with depression than healthy people (Xie et al., 2018).

It is also common for a person to experience multiple types of CM, and multiple maltreatment had more serious effects on individuals. Studies have shown that multiple maltreatment experiences lead to significantly more severe depressive symptoms and are important predictors of symptom severity in patients with chronic depression (Negele et al., 2015). Comparing the prevalence of three or more, four or more, and five types of CM, the results shows that the prevalence with multiple maltreatment was higher among MDD sample than non-clinical sample, suggesting that multiple maltreatment was more common among MDD patients than healthy adolescents, which deserves our attention and further highlights the importance of screening for CM in MDD patients. Neglect (physical and/or emotional) was reported more frequently than abuse (emotional and/or physical) in both samples, which is similar to the previous research on the prevalence of CM among Chinese adolescents and patients with depression (Xie et al., 2018; Yu et al., 2020). Conversely, according to a meta-analysis of the global prevalence of CM (Stoltenborgh et al., 2015), childhood abuse is more prevalent than childhood neglect in North America. This difference may be related to cultural differences in family rearing styles and people’s cognitive understanding of the concepts of abuse and neglect. Notably, in a study of mothers’ perceptions of child abuse and neglect in nine countries, Chinese mothers had significantly higher thresholds for marking behaviors as abuse (i.e., fewer behaviors were considered abusive) than mothers in all other countries (Mesman et al., 2020).

Regarding reliability, our finding of a moderate and significant correlation of CTQ-SF scores in the first test and re-test 4 weeks later is similar to findings in previous reports (Kim et al., 2011, 2013). Likewise, our reliability data indicating that all CTQ-SF subscales except Physical Neglect met our internal consistency acceptability criteria in both samples are similar to previous findings indicating that Physical Neglect has a poor consistency (Gerdner and Allgulander, 2009; Kim et al., 2011, 2013). Gerdner and Allgulander (2009) suggested that this problem may reflect ambiguity in the structural theory of neglect, which encompasses neglect of care and supervision, as well as emotional and Physical Neglect. Based on the argument that the distinction between the concepts of Emotional Neglect and Physical Neglect is relatively vague, and that item 2 and item 26 in the CTQ-SF may represent emotional rather than Physical Neglect, Gerdner and Allgulander (2009) proposed an alternative five-factor model. Subsequent studies with Korean psychiatric patients and Nigerian adolescents also supported the supposition of heterogeneity of the Physical Neglect subscale (Kim et al., 2011; Aloba et al., 2020). Regarding the present work with Chinese respondents specifically, it is worth noting that item 26 of the Chinese version of the CTQ-SF has semantic ambiguity that could confound people’s understanding. Altogether, these considerations suggest that total scale and subscale have adequate reliability in both two samples except Physical Neglect which may need to be adjusted.

About the measurement error of this scale, the estimates of SEM were 4.48, and MDC_95_ were 12.41, indicating that when the CTQ-SF score changed by more than 13 points, there was a real change at the 95% confidence level. According to the scatter chart, we can find that some data points fall outside the consistency interval, indicating that there is a certain measurement error in the scale, which suggests that we need to be cautious when using the scale to compare differences and make a conclusion. We should pay attention to the actual situation and make a judgment after comprehensive consideration (Peeters, 2016).

In both samples in this study, subscale scores correlated more strongly with CTQ-SF total scores than with one another, indicating that each subscale is relatively independent, while being significantly related to the total scale, which suggests that the CTQ-SF performed with great structural validity in the two samples, similar to previous studies (Gerdner and Allgulander, 2009; Kim et al., 2013; He et al., 2019). Moreover, given that depression and anxiety symptoms have previously been associated with CM, and evidence indicating that CM is a risk factor for the development of anxiety and depression (Hovens et al., 2009; Gardner et al., 2019), the present study observed significant correlations of CTQ-SF scores with BDI and STAI scores shows the good concurrent validity of the CTQ-SF. According to the results of MRA, in the two samples, Emotional Abuse and Emotional Neglect have a significant positive prediction effect on depression, Emotional Abuse has a significant positive prediction effect on anxiety. In addition, in non-clinical sample, Emotional Neglect have a significant positive prediction effect on anxiety, Sexual Abuse, Physical Neglect have a significant positive prediction effect on depression and anxiety. Considering that the Cohen’s *f*^2^ of Sexual Abuse and Physical Neglect are very small, so the results mainly reveal the close relationship between Emotional Abuse, Emotional Neglect with depression and anxiety, which is consistent with previous studies (Infurna et al., 2016).

The present CFA results affirm the suitability of the five-factor model of CTQ-SF in non-clinical and MDD samples. Specifically, acceptable fit indexes were obtained without any modifications in the non-clinical sample and its subgroup of females, whereas achieving a good fit with the MDD sample and other groups could be achieved with a modification. These results are consistent with the results of previous studies carried out in samples in other countries, including Italian, Norwegian, and Danish samples (Dovran et al., 2013; Sacchi et al., 2017; Kongerslev et al., 2019), in indicating that the five-factor structure of the CTQ is retained across cultural contexts.

Due to the low consistency of the Physical Neglect subscale, we also examined an alternative five-factor model proposed by Gerdner and Allgulander (2009) (results reported in Supplementary Material). In the alternative model, two items of the original questionnaire (#2 – “I knew there was someone to take care of me and protect me” and #26 – “There was someone to take me to the doctor if I needed it) are instead loaded into the Emotional Neglect scale, leaving only three items in the Physical Neglect subscale, while the Emotional Neglect subscale is enlarged to seven items, and the other scales remain unchanged. Gerdner and Allgulander validated their alternative five-factor model in seven samples (five clinical and two NC) in Switzerland. The alternative five-factor model was also found to be more suitable than the original model in studies of Korean psychiatric patients and Nigerian adolescents (Kim et al., 2013; Aloba et al., 2020). Here, because there was no significant difference between the results obtained for the two models and the original model is more widely recognized, we chose to maintain the original model for measurement equivalence testing.

Scalar invariance of the CTQ-SF across genders was demonstrated previously in both Nigerian adolescents and Chinese college students (He et al., 2019; Aloba et al., 2020), and partial weak equivalence was demonstrated previously across genders among drug-abusing adults (Meredith, 1993). However, in a US study, the only structural equivalence of the CTQ-SF was supported across genders (Rodriguez et al., 2018). These findings remind us that it is necessary to evaluate the measurement invariance of CTQ-SF among non-clinical and MDD samples. The present measurement invariance testing established configural, metric, and scalar invariance of the CTQ-SF across genders within the non-clinical sample as well as across the non-clinical sample and MDD sample, with a demonstration of scalar invariance across test and 4-week retest time points. Thus, the present data suggest that the meaning of the CTQ-SF concept is maintained across gender, time, and presence of depression which means the CTQ-SF scores are comparable between groups.

The expectation that MDD patients may have more severe CM in their personal histories than healthy adolescents is typically supported by the present findings of the MDD sample having higher CTQ-SF total scores and subscale scores than that were obtained for the non-clinical sample, except for the Sexual Abuse subscale score. Previously, higher CTQ scores across all subscales have been associated with depression in German samples (Christoph et al., 2016; Kaczmarczyk et al., 2018). Regarding differences across samples, it may be relevant that traditional Chinese culture is sensitive to sexual topics, which could lead to underreporting and concealment of the occurrence of Sexual Abuse. In terms of the gender differences of CTQ-SF scores, our study found that in non-clinical sample, boys were significantly higher than girls in all dimensions except emotional abuse, which was consistent with the results of previous studies on Chinese college students (He et al., 2019). However, due to the small effect sizes of Sexual Abuse and Emotional Neglect, the main differences between male and female students were in Physical Neglect and Physical Abuse, which suggests that boys suffer more severe Physical Neglect and Physical Abuse than girls. This result is partly related to the different parenting styles for boys and girls in Chinese culture, in which parents’ expectations for girls are influenced by the chastity virtue (Shek, 2008). Compared to boys, parents tend to exert higher behavioral control and foster dependency in girls. Also, the bond between parents and daughters tends to be stronger, and girls are experience less physical abuse or neglect than boys (Lo et al., 2018, 2019). In the MDD sample, boys scored significantly higher than girls in emotional neglect, and there was no significant difference in other dimensions. It shows that boys with MDD are more serious than girls in Emotional Neglect.

This study has several limitations which should be taken into account in interpreting the results. Moderate-sized convenience samples were recruited entirely from within Hunan province in China. Thus, the applicability of the results obtained with the Chinese version of the CTQ-SF to the whole of China and Chinese speakers outside of China cannot be assumed. In addition, since we did not retest the depression sample and the sample size was small, we could not explore the measurement invariance across gender and time among the MDD sample, which also limited analyzing measurement invariance in MDD sample that combined horizontal and longitudinal perspectives. It’s worth noting that because of the clinical sample was restricted to only participants with a single diagnosis of MDD, our results are not generalizable to the patients with other psychiatric diagnoses (e.g., PTSD) and the MDD patients which have a psychiatric comorbidity, and there remains a need to examine the presently addressed questions in more varied clinical samples. Finally, this study relied on self-reported CTQ-SF results. Thus, the data could have been affected by underreporting of CM by participants.

## Conclusion

The scope of scale application for the CTQ-SF was expanded with this study. The CTQ-SF was demonstrated to be an effective and reliable tool for assessing CM in Chinese adolescents. Measurement invariance of the CTQ-SF was established across genders and the presence of depression, as well as over time, in Chinese adolescents, indicating that the Chinese version of CTQ-SF can be meaningfully used to compare outcomes across genders, time points, and the presence of depression among Chinese adolescents.

## Data Availability Statement

The raw data supporting the conclusions of this article will be made available by the authors, without undue reservation.

## Ethics Statement

The studies involving human participants were reviewed and approved by the Ethics committee of Second Xiangya Hospital, Central South University. Written informed consent to participate in this study was provided by the participants’ legal guardian/next of kin.

## Author Contributions

SY and XiaW contributed to conception, design, and supervise the study. XinW and FD performed the statistical analysis and wrote the first draft of the manuscript. CC and JH collected the data and contributed to the analysis. All authors contributed to manuscript revision and approved the submitted version.

## Conflict of Interest

The authors declare that the research was conducted in the absence of any commercial or financial relationships that could be construed as a potential conflict of interest.

## Publisher’s Note

All claims expressed in this article are solely those of the authors and do not necessarily represent those of their affiliated organizations, or those of the publisher, the editors and the reviewers. Any product that may be evaluated in this article, or claim that may be made by its manufacturer, is not guaranteed or endorsed by the publisher.
