# Supplementary material for: Psychometric Properties and Measurement Invariance of the Childhood Trauma Questionnaire (Short Form) Across Genders, Time Points and Presence of Major Depressive Disorder Among Chinese Adolescents
Source: Front Psychol. 2022 Apr 11;13:816051. doi: 10.3389/fpsyg.2022.816051 (PMC9036057; doi:10.3389/fpsyg.2022.816051)
Supplement: Supplementary file 1 [file Table_1.DOCX]

**Table 1** CFA for the original and alternative five-factor CTQ-SF models

| **Model and sample** | **S-Bχ2** | **CSF** | **df** | **CFI** | **TLI** | **RMSEA(90%CI)** | **SRMR** |
| --- | --- | --- | --- | --- | --- | --- | --- |
| Original model |  |  |  |  |  |  |  |
| Non-clinical sample | 675.333* | 2.199 | 265 | 0.914 | 0.903 | 0.032(0.029-0.035) | 0.049 |
| MDD sample | 466.965* | 1.466 | 265 | 0.888 | 0.874 | 0.052(0.044-0.060) | 0.066 |
| MDD_modified_ | 418.796* | 1.443 | 264 | 0.914 | 0.903 | 0.046(0.037-0.054) | 0.066 |
| Alternative model |  |  |  |  |  |  |  |
| Non-clinical sample | 626.179* | 2.163 | 265 | 0.925 | 0.915 | 0.030（0.027-0.033） | 0.041 |
| MDD sample | 417.297* | 1.463 | 265 | 0.886 | 0.871 | 0.053（0.045-0.060） | 0.065 |
| MDD_modified_ | 423.128* | 1.440 | 264 | 0.912 | 0.900 | 0.046（0.038-0.054） | 0.065 |

*Note.* *p <0.05. MDD, major depressive disorder, MDD_modified_ means the model after error covariances. Bχ2, Chi-square test of model fit; CSF: Scaling Correction Factor for MLM; df, degrees of freedom; CFI, comparative fit index; TLI, Tucker-Lewis index; RMSEA, root mean square error of approximation; SRMR, standardized root mean square residual; CI, confidence interval.
